# Empiric Treatment for Suspected Malaria in the United States: A Case Report

**DOI:** 10.7759/cureus.7885

**Published:** 2020-04-29

**Authors:** Jonathan C Li, Emma Lundsmith

**Affiliations:** 1 Internal Medicine-Pediatrics, ChristianaCare, Newark, USA; 2 Internal Medicine, Thomas Jefferson University Hospital, Philadelphia, USA

**Keywords:** malaria, diagnostic accuracy, travel medicine, vivax infections, undiagnosed fever

## Abstract

Malaria in the United States is rare and most commonly presents among returning travelers from endemic areas. Diagnosis is classically dependent on a positive blood smear or polymerase chain reaction (PCR) test. The objective of this case report is to highlight a case of suspected malaria in a high-risk individual with negative diagnostic testing where a trial of empiric treatment was initiated based on clinical presentation after a thorough discussion of risks and benefits. However, empiric treatment based on a single case is limiting. We present a case of a 56-year-old man with extensive travel history throughout Asia, who presented after multiple episodes of unprovoked 24-hour fevers over the past seven years. A thorough rheumatologic and infectious inpatient workup was negative and oncology was consulted with low suspicion for malignancy. However, based on clinical presentation and history, malaria remained highly suspected and an empiric trial of anti-malarial treatment was initiated. One year after receiving treatment, the patient has not experienced any further febrile episodes. The efficacy of blood smears and PCR may be influenced by the malarial strain, as some species have low circulating biomass. Therefore, blood smears and PCR testing may not always be diagnostic. Clinical signs supportive of a malarial infection include fever, rigors, chills, hepato/splenomegaly, hyperbilirubinemia, and thrombocytopenia. Malaria is endemic to many regions outside of Africa, including Asia, and should be considered in any returning traveler with recurrent fevers.

## Introduction

Globally, malaria is a common cause of recurring fevers and is recognized to have high endemicity in sub-Saharan Africa and parts of Asia. However, malaria is also common outside of Africa and presents similarly but has distinct clinical differences. Blood smears with Giemsa stain are the gold standard for diagnosis [1]. However, some species of Plasmodium are less detectable in peripheral blood, and negative blood smears do not rule out the diagnosis [2,3].

## Case presentation

A 56-year-old man presented to the emergency department (ED) after experiencing a fever to 101°F (38.3°C) at home. His past medical history included intermittent febrile episodes over the past seven years and hypertension. His only medication was hydrochlorothiazide.

He experienced recurrent unprovoked febrile episodes (occurring every two to three months) for the past seven years. These episodes were acute in onset, without preference for day or night, and followed a stereotypic pattern. They began with chills/rigors followed by high-grade fever, severe day/nighttime diaphoresis, nausea, and sometimes palpitations. Spontaneous resolution typically occurred within 24-48 hours and left him feeling severely fatigued the following day. The highest fever he recorded during an episode was 104.1°F (40.1°C). He denied vomiting, diarrhea, abdominal pain, dysuria, arthralgias, myalgias, or unexpected weight loss in association with these episodes. Between episodes, he felt completely asymptomatic.

Five years prior to presentation, the patient resided in China for seven years. He also endorsed travel history to Taiwan, Malaysia, Thailand, Philippines, Korea, Japan, Costa Rica, and the Dominican Republic. He reports taking appropriate preventive measures against mosquitoes such as barriers and chemical prophylaxis while traveling. His first febrile episode occurred in Taiwan within the seven-year period when he was residing in China. Initially, after returning from China, he experienced dyspnea and chest pain with a negative cardiac and pulmonary workup.

He did not endorse any illicit drug use, tobacco use, and seldomly consumed alcohol. He was up to date with his preventive health screening exams. Family history was significant for rheumatoid arthritis in his sister. There was no other history of autoimmune disease or familial fever syndromes in his family.

The patient was admitted for systemic inflammatory response syndrome (SIRS) and for a diagnostic assessment for fever of unknown origin. Diagnostic labs and imaging are summarized in Table 1 and Table 2, respectively. Many of these were repeated during periods of fever without a significant difference in results. An extensive rheumatologic and infectious workup was negative and oncology was consulted with low suspicion for malignancy. The infectious disease service was consulted and reported concern for malaria (particularly *Plasmodium vivax* (*P. vivax*) and *Plasmodium ovale* (*P. ovale*)) and brucellosis due to his travel history, although there lacked typical periodicity. They noted that splenomegaly, anemia, thrombocytopenia, and mild transaminitis seemed consistent with malaria. A malaria expert was consulted at the Centers for Disease Control and recommended empiric treatment for malaria based on the patient’s history and clinical findings. Following a negative glucose-6-phosphate dehydrogenase (G6PD) deficiency screen, the patient was started on empiric treatment with oral atovaquone/proguanil 1000/400 mg for three days and primaquine 30 mg daily for 14 days.

**Table 1:** Blood tests Rheumatologic workup was negative for anti-Smith antibody, anti-SS-A antibody, anti-SS-B antibody, antinuclear antibodies (ANA), anti-dsDNA antibody, and rheumatoid factor. Infectious workup was negative for anaplasmosis, babesiosis, erlichiosis, malaria, Lyme, syphilis, tuberculosis, hepatitis B, hepatitis C, HIV, cytomegalovirus (CMV), and Epstein-Barr virus (EBV).

Lab	Measured value	Reference range
High sensitivity troponin	< 6 (N)	< 19 ng/L
D-dimer	434 (H)	< 204 ng/mL
International normalized ratio (INR)	1.23 (N)	0.8-1.19
Prothrombin time (PT)	13.6 (H)	8.9-13.1 sec
Partial thromboplastin time (PTT)	24 (N)	24-35 sec
Urinalysis	Unremarkable	
Urine drug screen	Negative	
Urine culture	Negative	
White blood cell	7.6 (N)	4.0-11.0 B/L
Neutrophil	91.40%	
Lymphocytes	5.00%	
Monocytes	2.90%	
Eosinophils	0.30%	
Basophils	0.10%	
Red blood cell	4.13 (L)	4.5-6 T/L
Hemoglobin	13.3 (L)	14-17 g/dL
Platelet	115 (L)	140-400 B/L
Blood cultures	Negative	
Blood smear	Unremarkable	
Lactate	2.6 (H)	0.5 - 2.0 mmol/L
C-reactive protein	0.30 (N)	≤ 0.80 mg/dL
Erythrocyte sedimentation rate	3 (N)	0-20
Thyroid stimulating hormone	1.31 (N)	0.3-5.00 uIU/mL
Lactate dehydrogenase	162 (H)	125-240 IU/L
Haptoglobin	75 (N)	16-200 mg/dL
Total bilirubin	1.3 (H)	1-1.2 mg/dL
Aspartate aminotransferase	45 (H)	0-40 IU/L
Alanine aminotransferase	76 (H)	0-44 IU/L
Alkaline phosphatase	56 (N)	39-117 IU/L
Iron	28 (L)	55-160 mcg/dL
Total iron binding capacity	247 (L)	250-400 mcg/dL
Iron saturation	11% (L)	20-55%
Ferritin	839 (H)	30-40 ng/mL
Anti Smith Antibody	8	0 - 89 U/ml
Anti SS-A Antibody	19	0 - 91 U/ml
Anti SS-B Antibody	1	0 - 73 U/ml
ANA screen	Negative	
Anti dsDNA Antibody	5	0 - 40 IU
Rheumatoid Factors	11	<14 IU/mL
A. phagocytoph. IgG	<1:64	<1:64
A. phagocytoph. IgM	<1:20	<1:20
Ehrlichia chaffeensis IgG	<1:64	<1:64
Malaria Antigen Screen	Presumptive Negative	
Lyme Ab screen	1.75 (equivocal)	<0.90 Index
Lyme Disease Ab (IgM)	Negative	
Lyme Disease Ab (IgG)	Negative	
Syphilis Antibody	Negative	
Quantiferon Result	Negative	
Tuberculosis Antigen	≤ 0.000	
Hep B Surf Antigen	Non-reactive	
Hep C Antibody	Non-reactive	
HIV Ab/Ag Result	Non-reactive	
Cytomegalovirus polymerase chain reaction (PCR), Plasma	<100 IU/mL	<100 IU/mL
Ebstein-Barr virus quantitative PCR, Plasma	Non detected	
Mono Screen	Non-reactive	
Babesia microti DNA reverse transcriptase-PCR	Not detected	
Babesia microti DNA PCR	Not detected	

**Table 2:** Imaging findings

Imaging	Findings	
Chest X-ray	Unremarkable	
Computed tomography (CT) angiography of pulmonary embolism (PE)	Negative for PE, mild interlobular interstitial thickening possibly representing pulmonary edema. Cardiac morphology and pericardium were unremarkable.	
CT abdomen pelvis	Mild splenomegaly measuring 16 x 12 cm, trace amounts of free fluid in the lower pelvis of uncertain etiology, and an enlarged lymph node near the liver.	

Three weeks following discharge, the patient was seen for outpatient follow-up. The anemia was resolving with a hemoglobin of 13.3 g/dL; thrombocytopenia fully resolved with a platelet count of 169 B/L. Mild transaminitis persisted (aspartate aminotransferase (AST) 42 IU/L, alanine aminotransferase (ALT) 64 IU/L), total bilirubin had returned to normal (0.7 mg/dL), and alkaline phosphatase (ALP) remained normal at 58 IU/L. Though the improvement in labs may have simply reflected the resolution of SIRS, the patient reported no additional fevers during a telehealth follow-up one year later.

## Discussion

The most common causes of recurrent febrile episodes include infectious, rheumatologic, and oncologic etiologies. Oncology did not suspect malignancy as the febrile episodes were too far inconsistent, complete blood cell count (CBC) and smear were normal, and the patient was up to date on cancer screening without a high-risk family history. Rheumatology had low suspicion for an autoinflammatory disorder as the patient had a lack of arthralgias and rash, was completely asymptomatic between episodes, and workup for common autoimmune disorders were negative. Finally, workup for common infectious etiologies including viral, bacterial, and parasitic were negative. Despite this, based on the patient's travel history and clinical presentation, malaria was still highly suspected [4].

There are five Plasmodium species known to infect humans: falciparum, malariae, ovale, vivax, and knowlesi [1]. *Plasmodium falciparum (P. falciparum*) is the most common, accounting for 99% of known malaria cases, and generally is geographically-limited to sub-Saharan Africa [2,5]. *P. vivax* is the most common species outside of Africa and accounts for 50% of non-African cases in the Americas, Eastern Mediterranean region, and Asia [2,5]. In Asia, the burden of *P. vivax* has been reported to be as high as 80% of the total global burden [4]. Other species in this region include *Plasmodium knowlesi*,*P. ovale*,* and Plasmodium malariae* [2,6].

Due to the infrequency of febrile episodes in this case, a Plasmodium species capable of producing hypnozoites seems most likely. Only *P. vivax* is confirmed to have a latent state in the liver (hypnozoites) with the ability to remain dormant for up to two years after primary infection [3]. *P. ovale* is suspected to have a hypnozoite stage as well, however, the evidence for this is limited and controversial [6]. Both *P. vivax *and *P. ovale* have reported relapse periods consistent with our patient’s presentation, however, *P. ovale* less commonly produces clinical malaria and is more common in Africa than Asia [6,7]. *P. vivax* is not commonly seen in Africa due to the lack of the Duffy antigen on erythrocytes in the region [2].

The classic episode of *P. vivax* infection begins with a prodrome of headache, anorexia, malaise, myalgias, and gastrointestinal symptoms for one or more days followed by a remitting fever [2]. Subsequent paroxysmal episodes occur in response to the rupturing of schizont-infected red blood cells (RBCs) [2]. These episodes begin with a stage of chills and rigors that last for approximately one hour, followed by fevers peaking 1-3 hours after rigors subside, and defervescence accompanied by diaphoresis and fatigue [2]. In comparison to *P. falciparum*, *P. vivax* induces an inflammatory response at a significantly lower parasite load [2]. Additionally, *P. vivax* has a predilection for reticulocytes, ultimately infecting < 2% of circulating erythrocytes while maintaining a greater capacity of causing severe anemia than *P. falciparum* [2]. The frequency of relapse depends on the infecting geographic strain of *P. vivax* [2]. Tropical strains relapse more frequently, from 1-6 months, and temperate strains relapse at intervals of eight months or greater [2].

Clinical signs and abnormalities

D-dimer is often elevated during malarial infections [8]. *P. falciparum* is well known to cause adherence of infected RBCs to the endothelium, causing damage and activation, and subsequently elevating D-dimer levels [8]. *P. vivax* is reported to have the same effect albeit with a 10-fold lower capacity. D-dimer levels in-turn reflect these pathophysiological differences [8].

In our case, our patient had a mild transaminitis and mild hyperbilirubinemia. Severe malarial infections can cause “malarial hepatopathy” which are defined by severe elevations of liver function tests [9]. These serologic elevations are higher in *P. falciparum *than *P. vivax* infections, suggesting that the etiology is more likely related to falciparum-specific etiologies (e.g. increased RBC cytoadherence) and not the presence of hepatic hypnozoites in *P. vivax* [9,10]. Our patient did not meet the criteria for malarial hepatopathy. We observed mild splenomegaly in our patient, a highly specific finding of malarial infection, and may simply reflect normal splenic filtration of abnormal RBCs, vascular congestion due to malaria, and organ-specific immune response against malaria [10,11].

On CT, our patient was found to have mild pulmonary edema and free peritoneal fluid. These findings are rarely reported in non-severe malarial infections and consistent with the effects that malaria exerts on endothelium and microvascular function [8,12].

Thrombocytopenia commonly occurs in malaria as well. The exact mechanism is not completely understood but centers around immunologic mechanisms damaging thrombocytes and causing excess platelet removal [13]. Thrombocytopenia is reported to have high sensitivity (94%), high specificity (73%), and a high negative predictive value (97%) for malaria [14].

Diagnostic studies

Analysis of thick smear blood samples under light microscopy has greater sensitivity for diagnosing *P. falciparum *than *P. vivax* due to *P. vivax* having a preference for infecting pre-circulating reticulocytes [3,15]. Serologic testing for lactate dehydrogenase (LDH) and aldolase is useful but requires a moderately elevated parasitemia [3]. Antigen-detecting rapid diagnostic tests (RDTs) are now one of the most prevalent diagnostic tools used worldwide reaching areas where microscopy and polymerase chain reaction (PCR) are limited [16]. Overall, PCR testing tends to be the most sensitive as it is able to assess the presence of malaria, even with low parasite counts [17]. We identified only one other report of malaria with negative PCR [18]. In settings where laboratory testing is limited or high suspicion is not corroborated by diagnostic serologies (as in our case), other clinical findings have consistently been shown to support a diagnosis of malaria. Positive predictors include fever, non-pulsatile headache, chills and rigor, and periodicity to these symptoms [19,20]. Negative predictors include conjunctival suffusion, rash, respiratory rate >28, severe arthralgia/myalgia, throat congestion, fever >40 C°, and continuous fever [19,20]. A previous study reports that among travelers returning to the U.S, the most supportive clinical findings for malaria were splenomegaly, hepatomegaly, hyperbilirubinemia, and thrombocytopenia [10]. From this case, additional predictors may include imaging findings of non-specific fluid shifts (pulmonary edema, peritoneal fluid) reflecting malaria-induced endothelial dysfunction and hepatic lymphadenopathy reflecting hepatic hypnozoite activity.

When the malaria species is unknown, the World Health Organization recommends treatment under the assumed diagnosis of uncomplicated malaria by *P. falciparum*. This means that artemisinin-based combination therapies should be primarily utilized, mainly for its ability to overcomes chloroquine-resistance. If the patient has G6PD, treatment should be centered around primaquine. Additional treatment with primaquine for 14 days in all transmission settings should be performed to prevent relapse [1].

## Conclusions

Malaria is common and endemic to many countries outside of Africa (e.g. Asia), with *P. vivax* as the predominating, causative species. PCR is the best test when suspicious for *P. vivax*, however, it is not always diagnostic. Recurrence of febrile episodes maybe months to years apart. Clinical signs supportive of a malarial infection are fever, rigors, chills, hepato/splenomegaly, hyperbilirubinemia, and thrombocytopenia. Severe manifestations of malaria are life-threatening. G6PD should be tested for prior to empiric treatment. When the malarial organism is unknown, treatment is approached as if the patient has an uncomplicated *P. falciparum* infection.

## References

[1] (2019). World Health Organization: guidelines for the treatment of malaria. https://apps.who.int/iris/bitstream/handle/10665/162441/9789241549127_eng.pdf;jsessionid=009E8121020BEEEC28E783F8F4C01DD1?sequence=1.

[2] Anstey NM, Douglas NM, Poespoprodjo JR, Price RN (2012). Plasmodium vivax: clinical spectrum, risk factors and pathogenesis. Adv Parasitol.

[3] Dayananda KK, Achur RN, Gowda DC (2018). Epidemiology, drug resistance, and pathophysiology of Plasmodium vivax malaria. J Vector Borne Dis.

[4] Kallinich T, Gattorno M, Grattan CE (2013). Unexplained recurrent fever: when is autoinflammation the explanation?. Allergy.

[5] (2019). World Health Organization: world malaria report 2017. https://apps.who.int/iris/bitstream/10665/259492/1/9789241565523-eng.pdf.

[6] Groger M, Fischer HS, Veletzky L, Lalremruata A, Ramharter M (2017). A systematic review of the clinical presentation, treatment and relapse characteristics of human Plasmodium ovale malaria. Malar J.

[7] Mueller I, Zimmerman PA, Reeder JC (2007). Plasmodium malariae and Plasmodium ovale-the “bashful” malaria parasites. Trends Parasitol.

[8] Meltzer E, Keller S, Shmuel S, Schwartz E (2019). D-dimer levels in non-immune travelers with malaria. Travel Med Infect Dis.

[9] Woodford J, Shanks GD, Griffin P, Chalon S, McCarthy JS (2018). The dynamics of liver function test abnormalities after malaria infection: a retrospective observational study. Am J Trop Med Hyg.

[10] Taylor SM, Molyneux ME, Simel DL, Meshnick SR, Juliano JJ (2010). Does this patient have malaria?. JAMA.

[11] Buffet PA, Safeukui I, Deplaine G (2011). The pathogenesis of Plasmodium falciparum malaria in humans: insights from splenic physiology. Blood.

[12] Barber BE, William T, Grigg MJ (2015). Parasite biomass-related inflammation, endothelial activation, microvascular dysfunction and disease severity in vivax malaria. PLoS Pathog.

[13] Naing C, Whittaker MA (2018). Severe thrombocytopaenia in patients with vivax malaria compared to falciparum malaria: a systematic review and meta-analysis. Infect Dis Poverty.

[14] Arshad AR (2015). Thrombocytopenia in malaria: can platelet counts differentiate malaria from other infections?. J Coll Physicians Surg Pak.

[15] Stauffer WM, Cartwright CP, Olson DA (2009). Diagnostic performance of rapid diagnostic tests versus blood smears for malaria in US clinical practice. Clin Infect Dis.

[16] (2018). Summary results of WHO product testing of malaria RDTs: round 1-8 (2008-2018).

[17] Mfuh KO, Achonduh-Atijegbe OA, Bekindaka ON (2019). A comparison of thick-film microscopy, rapid diagnostic test, and polymerase chain reaction for accurate diagnosis of Plasmodium falciparum malaria. Malar J.

[18] Bhome R, Bhome R (2011). PCR negative cerebral malaria in a traveller returning from Mumbai. BMJ Case Rep.

[19] Shah V, Shah BK, Vadera B, Acharya HK (2011). Clinical scoring system to predict malarial fever: a prospective study. Int J Med Public Health.

[20] Chandramohan D, Carneiro I, Kavishwar A, Brugha R, Desai V, Greenwood B (2001). A clinical algorithm for the diagnosis of malaria: results of an evaluation in an area of low endemicity. Trop Med Int Health.

